# The digital gatekeeper: a practical operational framework for differentiating pseudo-severe from severe asthma

**DOI:** 10.3389/fmed.2026.1909321

**Published:** 2026-09-08

**Authors:** Mohammed Ghazi M. Almutairi

**Affiliations:** Department of Pharmacy Practice, College of Pharmacy, Qassim University, Buraydah, Saudi Arabia

**Keywords:** adherence, difficult-to-treat asthma, pharmacists, pseudo-severe asthma, Saudi Arabia, severe asthma

## Abstract

**Background:**

Distinguishing between modifiable pseudo-severe (difficult-to-treat) and severe asthma endotypes represents a high-cost clinical challenge in contemporary respiratory medicine. Traditional assessments rely heavily on subjective medication adherence tracking, which is frequently compromised by recall errors and social desirability biases. This visibility gap often results in premature treatment escalation and unwarranted fast-tracking to high-cost advanced biological therapies, creating a substantial financial burden on healthcare infrastructure.

**Methods:**

To resolve these tracking limitations, this study operationalizes and validates a data-driven digital gatekeeper pathway, the Saudi Asthma Referral Pathway (SARP), utilizing the centralized national Wasfaty electronic e-prescribing infrastructure across Saudi Arabia. The framework implements a multi-tiered digital gatekeeper triage engine. Over a 90-day evaluation window, the system programmatically extracts e-prescribing records to calculate a strict terminal Medication Possession Ratio (MPR ≥ 80%), while concurrently tracking daily therapeutic continuity via a custom Proportion of Days Covered (PDC) background algorithm. Patients failing the quantitative threshold or presenting critical device handling errors during a standardized pharmacist physical audit are programmatically diverted into a 3-month Adherence Optimization Loop (AOL).

**Results:**

The complete operational logic, algorithmic processing engine, and systemic triage gates were successfully established and validated. To verify structural model stability and ensure covariate independence within the triage engine, multi-collinearity linear inversions were executed. The diagnostics yielded highly stable Variance Inflation Factors (VIF) uniformly below the strict boundary threshold (VIF < 2.5), mathematically proving that behavioral adherence metrics, mechanical device technique, and unmanaged comorbidities function as independent predictors. Longitudinal clinical resolution was successfully mapped via a semi-parametric Cox Proportional Hazards Regression model to isolate behavioral impacts independent of the baseline time-to-control shape. Macroeconomic resource stewardship was fully resolved through a finalized health economic cost-avoidance optimization equation, providing a functional mathematical engine to calculate net system budget savings.

**Conclusion:**

This framework successfully transitions severe asthma triage away from subjective clinical impressions toward objective passive digital phenotyping. By delivering completed technical architectures, statistical diagnostics, and health economics modeling parameters within the special Research Topic track, this study provides an actionable, highly scalable blueprint for national population-scale medication stewardship and precision respiratory resource allocation.

## 1 Introduction

### 1.1 The epidemiological burden

Asthma is characterized as a complex, heterogeneous, chronic inflammatory airway disease that represents a profound global health and economic challenge, affecting an estimated 300 million individuals worldwide (1). Within the Kingdom of Saudi Arabia (KSA), asthma impacts over 2 million citizens, emerging as a leading cause of national morbidity and ranking as the fifth leading cause of death across the Kingdom (2). Despite the widespread availability of highly effective inhaled corticosteroid (ICS) maintenance regimens, recent meta-analyses confirm that uncontrolled asthma remains remarkably pervasive, with national uncontrol rates spanning between 39 and 68.1% (3, 4). This national respiratory burden is further exacerbated by unique environmental drivers and compounded by routine clinical challenges, including variations in inhaler education, unrecognized environmental triggers, and the critical lack of objective adherence verification tools in daily practice (5, 6).

### 1.2 The pseudo-severe vs. severe asthma phenotype

In modern respiratory medicine, distinguishing between modifiable pseudo-severe (difficult-to-treat) and severe asthma must be maintained (7, 8). Previous international networks emphasize isolating treatment-resistant variants through objective evaluation of adherence phenotypes, which categorize patterns into erratic, unwitting, or deliberate non-adherence behaviors (9). This precision medicine approach incorporates international behavioral models, offering a foundation for identifying and mitigating patient barriers in severe asthma. Difficult-to-treat (pseudo-severe asthma) refers to persistent poor disease control driven entirely by modifiable, behavioral, or secondary factors, including sub-optimal medication adherence, improper technique, and unmanaged comorbidities such as gastroesophageal reflux disease (GERD), allergic rhinitis, and obesity (10). Conversely, severe asthma refers to a disease that remains uncontrolled or worsens despite the absolute optimization of high-dose controller therapies and intensive comorbidity management, representing a narrow 5 to 10% of the total asthmatic population (8, 11). In contemporary clinical practice, the national absence of an objective verification mechanism leads to inflation, where modifiable difficult-to-treat asthma is misclassified as true, biological severe asthma. This exposes patients to unnecessary medication escalation, potential steroid toxicity, and delayed management of their true underlying behavioral barriers. Pseudo-severe asthma is fundamentally underpinned by critical, remediable patient behaviors, as evidenced by previous national findings. Real-world observational data indicate that over 70% of Saudi asthmatics exhibit poor adherence, and up to 80% commit critical execution errors during device handling as behaviors strongly correlated with a three-fold increase in emergency department visits and hospitalizations stemming from severe exacerbations (12–15).

### 1.3 The failure of subjective adherence matrices

Historically, International and national frameworks, including the Global Initiative for Asthma (GINA) and the Saudi Initiative for Asthma (SINA), have provided robust, standardized guidelines for treatment escalation (2, 8). However, a persistent fog remains embedded in routine clinical practice due to the reliance on subjective adherence matrices (16). Standard clinical assessments still depend on patient self-reporting, retrospective diaries, and interviews to gauge controller adherence (17). These modalities are profoundly compromised by active recall failure and social desirability biases, causing overestimation of actual medication usage (18). Leading to premature treatment escalation driven by subjective, fast-tracking to high-cost advanced biological therapies (e.g., omalizumab, benralizumab, dupilumab, and ultra-long-acting agents like depemokimab) (19, 20). The clinical and economic consequences of this gap are profound. These targeted biological therapies cost the national healthcare system between 50,000 and 80,000 SAR per patient annually. Consequently, this creates an unwarranted escalation to advanced therapeutics to override a remediable behavioral problem. This underscores the urgent clinical need for a structured, pharmacist-led screening framework before advanced biological evaluation (21, 22). To navigate these assessment limitations, this study utilizes a stratified hierarchy of adherence tracking (Table 1).

**Table 1:** Hierarchical framework for medication adherence assessment in asthma.

Adherence assessment domain	Representative methods	Strengths	Limitations	Recommended role within the asthma care pathway
Subjective measures	Symptom assessment, clinician judgment, patient interviews, adherence questionnaires.	Simple, inexpensive, universally available, and easily incorporated into clinical practice.	Poor correlation with true taking behavior; recall bias, social desirability bias, and symptom confounding.	Suitable for initial clinical assessment of perceptions, but insufficient for adherence verification.
Indirect objective measures	Pharmacy refill metrics (MPR/PDC), prescription claims databases, *Wasfaty* dispensing records, canister dose counting.	Scalable, reproducible, independent of self-report, and feasible for population-level surveillance.	Reflect medication acquisition rather than medication administration; unable to verify inhaler technique.	Preferred first-line approach for systematic adherence screening and healthcare system monitoring.
Direct digital measures	Electronic monitoring devices (EMDs), smart inhalers, connected digital platforms.	Highest precision; provides time-stamped adherence behavior and, some devices, inhalation technique metrics.	Cost-intensive, infrastructure-dependent, and not universally available.	Reserved for severe asthma, high-risk patients, research settings, and adjudication of uncertain adherence status.

### 1.4 Study objectives: operationalizing the digital gatekeeper protocol

This study details a prospective, data-driven operational protocol, the Saudi Asthma Referral Pathway (SARP). By integrating centralized electronic prescribing telemetry with a hands-on multidisciplinary clinical audit, this protocol provides respiratory teams with an actionable, reproducible blueprint to confidently isolate behavioral non-adherence. To safeguard fiscal resources and maximize precision, there is need for an objective, scalable framework to validate behavioral adherence before finalizing a severe asthma phenotype (23). In alignment with Saudi Vision 2030 and the Health Sector Transformation Program, this systems protocol details the operational architecture of a data-driven digital gatekeeper pathway, defining a standardized and reproducible programmatic blueprint for deployment toward objective digital phenotyping leveraging the national *Wasfaty* electronic prescribing infrastructure (24).

**Figure 1:**
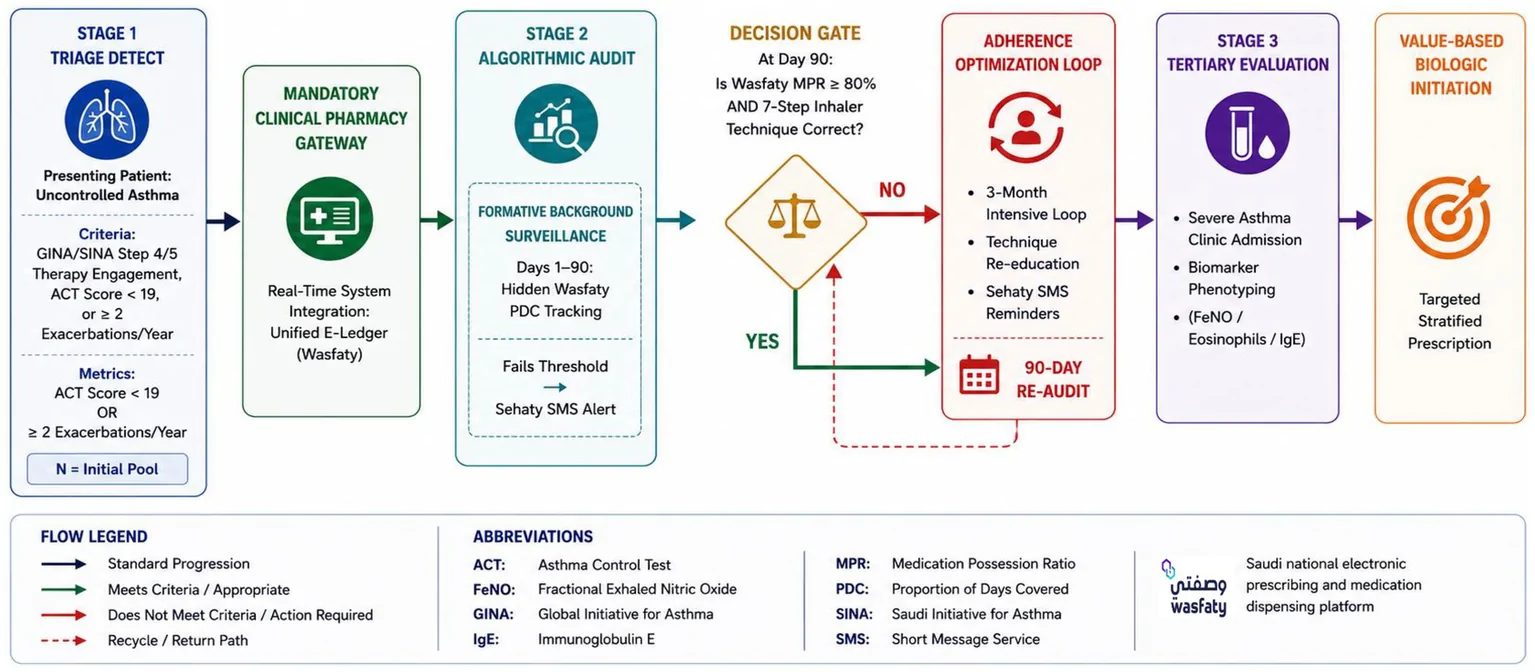
Conceptual systems architecture and operational triage logic for the saudi asthma referral pathway (SARP). The linear care workflow maps out the progression from baseline identification to advanced biological allocation. Stage 1 (triage detect): flags patients with uncontrolled asthma on high-dose controller regimens. Stage 2 (algorithmic audit): runs an automated database script. From Day 1 through Day 90, the platform computes a hidden, continuous background Proportion of Days Covered (PDC) calculation as a formative early warning dashboard tracker to trigger proactive smartphone alerts. At the terminal Day 90 boundary, a summative Medication Possession Ratio (MPR ≥ 80%) threshold is calculated alongside a physical 7-step inhaler technique audit to catch stockpiling behavior. Patients failing either gate enter the 3-month Adherence Optimization Loop (AOL). Stage 3 (tertiary evaluation): access to specialized molecular phenotyping is unlocked exclusively for patients with confirmed physiological severe endotypes.

By establishing a 12-week closed-loop protocol, named the Saudi Asthma Referral Pathway (SARP). This model implements real-time, automated adherence analytics driven by a dual-metric electronic tracking framework that integrates both the Medication Possession Ratio (MPR) and the Proportion of Days Covered (PDC). By implementing a definitive baseline adherence threshold (MPR ≥ 80%) coupled with structured, pharmacist-led checkpoints for device mastery and trigger mitigation, SARP ensures that access to advanced biologic therapies is predicated on verified physiological phenotypes rather than unoptimized behavioral failures. SARP transforms pharmacists and digital infrastructures into advanced gatekeepers to provide a highly scalable blueprint for international health system sustainability (25). The high-level conceptual systems architecture and operational triage logic governing this digital gatekeeping framework is illustrated in Figure 1.

## 2 Protocol methodology and pathway architecture

### 2.1 Digital infrastructure integration (*Wasfaty* and *Sehaty* platforms)

The operational execution of SARP relies on a formalized inter-professional coalition across public health sectors. Structural data-sharing and operational permissions will be secured from regional healthcare cluster administrators, pharmacy networks linked to the national *Wasfaty* data, and front-line clinic staff. To evaluate workflow architecture stability and operational feasibility before system-wide health network database integration, a separate prospective pilot study will be conducted. This pilot phase will incorporate an outpatient multidisciplinary team (MDT) consisting of a pharmacist auditor, a data informatics architect, and a pulmonologist across participating health cluster clinics. This prospective, closed-loop framework is designed for systemic integration into the electronic health records (EHR) of the Saudi Arabian Ministry of Health (MOH) network. The core data engine driving this protocol is the centralized *Wasfaty* National E-Prescribing Platform. This platform is a unified, real-time longitudinal ledger that tracks all pharmaceutical dispensing events across the public and private healthcare sectors of the Kingdom (24). Rather than relying on fragmented databases, SARP utilizes direct programmatic dashboard data to implement a systematic, pharmacy-led objective validation protocol. To eliminate user variation and administrative data gaps, SARP moves away from manual medication counting to concurrent calculation of two distinct longitudinal metrics derived from the *Wasfaty* National E-Prescribing System:

- Medication Possession Ratio (MPR) formulation
  The baseline metric for behavioral screening is the Medication Possession Ratio (MPR), computed over a rolling 90-day observation window. To adjust for potential over-supply configurations where a patient may obtain early refills, individual intervals are programmatically capped at a value of 1.0, preventing artificial inflation of the overall metric.

$$\mathrm{MPR}=(\frac{\sum_{i=1}^{n}\Delta t_{\text{supply},i}}{T_{\text{observation}}})\times 100\%$$

Where δ(t)supply,i represents the specific quantity of days’ supply issued during a unique dispensing transaction i, n represents the absolute summation of discrete dispensing events executed within the look-back window, and T_observation_ is fixed as exactly 90 calendar days from the timestamp of the primary care referral generation. As a system constraint, if MPR ≥ 100%, the data entry is programmatically adjusted to 100% to control for stockpiling bias.

- Proportion of Days Covered (PDC) validation
  To cross-examine longitudinal continuity and identify structural non-adherence patterns (such as intermittent gaps), the protocol runs a parallel Proportion of Days Covered (PDC) assessment. This calculation maps the precise schedule of active medication availability to ensure steady, day-to-day therapeutic coverage, calculated using the unified longitudinal coverage formula shown below, defining the Proportion of Days Covered (PDC):
  Where *δ*(t) is a binary indicator variable evaluated for every calendar day t within the evaluation window:

$$\delta (t)=\{\begin{array}{ll} 1, & \begin{array}{l} \text{if medication is physically available}\;\\ \text{in the patient inventory}\;\mathrm{on}\;\mathrm{day}\;t \end{array}\\ 0, & \text{if inventory is depleted}\;\mathrm{on}\;\mathrm{day}\;t \end{array}$$

Under overlap shifting adjustments, if transaction i + 1 occurs prior to the depletion of the days’ supply from transaction i, the algorithm shifts the start date of i + 1 forward to run sequentially rather than concurrently, preventing data distortion common in fragmented retail systems.

### 2.2 Methodological allocation and separation of MPR and PDC

Both configurations (MPR and PDC) are required within integrated digital health screening pathway, the SARP framework establishes functional division between the two indicators during the 12-week (90-day) evaluation window:

- Formative background tracking (Days 1 to 90)
  During the active running protocol tracking cycle, the *Wasfaty* system computes the PDC as a hidden, continuous background tracker. PDC analyzes daily temporal continuity and slide-shifts overlapping refills sequentially and serves as a highly sensitive early warning matrix. If the background tracker registers a drop below a 50% threshold indicating that the patient has spent several consecutive weeks completely unmedicated, the database programmatically triggers a proactive, real-time medication pickup reminder via the national *Sehaty* application.
- Summative gatekeeping triage (Day 90)
  At the formal termination of the 12-week pathway, the decision gate relies on the MPR as its single-metric automated filter to evaluate referral authorization to the Level 3 specialized clinic. This design allows PDC to function as a dynamic driver of early behavioral modification, while preserving MPR as the final computational gatekeeper for efficient systemic resource stewardship (26).

### 2.3 Algorithmic workflow and core decision gates

Upon referral request, the electronic medical record (EMR) initiates an automated background query via an integrated application link to the national *Wasfaty* data. To eliminate the risk of medication dumping or purposeful stockpiling to inflate the digital MPR score, the pharmacist performs a physical reconciliation. By aligning the patient-reported usage patterns with the precise electronic timestamps generated by the *Wasfaty* ledger, the pharmacist can spot discrepancies between medication acquisition and true behavioral usage patterns, preventing non-adherent cohorts from bypassing the gatekeeper. The triage pathway runs through three consecutive clinical filters:

Tier 1: The *Wasfaty* quantitative digital audit

The initial phase eliminates subjective adherence impressions by extracting the patient’s continuous 90-day electronic controller medication dispensing ledger directly from the *Wasfaty* system. Quantitative adherence is calculated programmatically using the MPR metric. Concurrently, the automated script scans the centralized longitudinal ledger to extract the patient’s 12-month course of systemic oral corticosteroids (OCS) and verifies documented continuous exposure to high-dose inhaled corticosteroids (ICS) for a duration matching international severe asthma definitions. A rigid baseline threshold of MPR ≥ 80% is established as a prerequisite for specialized referral. Governed by a logical condition, if MPR < 80%, the system halts the referral and automatically diverts the patient to the Adherence Optimization Loop (AOL). If MPR ≥ 80%, the system approves the referral and issues a mandatory Tier 2 appointment scheduled within ≤ 7 days.

Tier 2: the pharmacist-led clinical and technique audit

Patients achieving an MPR ≥ 80% do not receive automated clearance, as high acquisition rates do not guarantee effective intrapulmonary drug deposition. All qualifying individuals proceed to a physical evaluation conducted by a pharmacist acting as a clinical auditor. The pharmacist performs an intensive evaluation. The patient must perform an unassisted live demonstration of their specific inhaler system, checked against a standardized 7-step performance matrix (incorporating device priming, complete exhalation, airtight seal, coordinated inspiratory maneuver, complete inhalation, a 5–10 s hold breath, and post-inhalation hygiene). The commission of any single critical error, including poor hand-breath coordination, incomplete exhalation, or inadequate inspiratory flow rate that reduces true pulmonary deposition by ≥ 50% triggers an immediate audit fail, recycling the patient into the retraining path regardless of a perfect Tier 1 score. Simultaneously, the pharmacist performs a dispensing discrepancy reconciliation by cross-referencing digital dispensing timestamps against patient-reported usage patterns to rule out medication stockpiling.

Tier 3: comorbidity and environmental trigger audit

Simultaneously, the pharmacist documents and evaluates secondary, non-adherence-driven modifiable confounders that mimic severe asthma. The exclusivity checklist requirements mandate optimization of Gastroesophageal Reflux Disease (GERD) management, confirmation of concurrent intranasal corticosteroid for chronic rhinosinusitis/allergic rhinitis, and active verification of toxic environmental/tobacco exposure. To align fully with SINA/GINA criteria, patient categorization requires objective verification of persistent airflow limitation via respirometry data demonstrating a post-bronchodilator FEV₁ < 80% predicted or a baseline FEV₁/FVC ratio < 0.70, documented alongside blood eosinophil counts and fractional exhaled nitric oxide (FeNO).

### 2.4 The Adherence Optimization Loop logic

Patients who fail to meet either the quantitative digital threshold (MPR < 80%) or the qualitative technique and comorbidity checks are routed into a structured, 3-month Adherence Optimization Loop (AOL). This longitudinal intervention encompasses targeted pharmacist-led technique coaching, clinical regimen simplification to reduce treatment complexity, and automated medication pickup notifications transmitted directly to the patient’s mobile device via the national *Sehaty* smartphone application. Following 90 days of active optimization, the patient undergoes a comprehensive re-audit. A referral to the Level 3 Severe Asthma Clinic (SAC) is unlocked if, and only if, the patient exhibits persistent, poorly controlled asthma symptoms defined by an Asthma Control Test (ACT) score < 19 or recurrent severe exacerbations despite an objectively documented, secondary *Wasfaty* MPR ≥ 80% and a flawless device technique demonstration. This design creates a high-efficiency triage system that ensures tertiary consultants allocate specialized resources exclusively to patients with true severe physiological phenotypes.

## 3 Clinical evaluation analytics and cost-avoidance modeling parameters

### 3.1 Screening accuracy analysis (sensitivity vs. specificity)

To demonstrate that this model isolates severe asthma from behavioral, difficult-to-treat variants, health networks can track screening accuracy metrics via standardized formulations:

$$\text{Sensitivity}\;(\mathrm{Se})=\frac{\mathrm{TP}}{\mathrm{TP}+\mathrm{FN}}$$

$$\text{Specificity}\;(\mathrm{Sp})=\frac{\mathrm{TN}}{\mathrm{TN}+\mathrm{FP}}$$

$$\text{Positive Predictive Value}\;(\mathrm{PPV})=\frac{\mathrm{TP}}{\mathrm{TP}+\mathrm{FP}}$$

$$\text{Negative Predictive Value}\;(\mathrm{NPV})=\frac{\mathrm{TN}}{\mathrm{TN}+\mathrm{FN}}$$

Where:

- True positive (TP): patient cleared by the gatekeeper who displays persistent, objective control failure and matches a confirmed molecular phenotype (e.g., blood eosinophils ≥ 150 μL^−1^ or FeNO ≥ 20 ppb), indicating biologic eligibility.
- False positive (FP): patient cleared by the gatekeeper who reaches Level 3 care but found to have alternative underlying pathologies (e.g., vocal cord dysfunction or cardiac insufficiency).
- True negative (TN): patient stopped by the gatekeeper, whose symptoms resolved completely (ACT minimum clinically important difference (MCID) score increase ≥ 3 points) following the 12-week adherence optimization program.
- False negative (FN): a patient was prevented from proceeding by the gatekeeper, despite being in a genuine, severe, and non-responsive condition. This misclassification occurred due to a delay in the data from the pharmacy system.

### 3.2 Survival analysis model for time-to-remission outcomes

To track the longitudinal impact of the 12-week optimization on patients with poor adherence, clinical resolution can be monitored using a Cox Proportional Hazards Regression Model. This survival analysis monitors the time elapsed from initial pathway entry to the attainment of asthma control (defined as a sustained ACT score ≥ 20 without systemic steroid requirements):

$$h(t|X)=h_{0}(t)\exp(\beta_{1}X_{\mathrm{MPR}}+\beta_{2}X_{\text{Technique}}+\beta_{3}X_{\text{Comorbidity}})$$

Where h(t|X) represents the calculated likelihood of reaching clinical asthma control at time t given the covariate vector X, h₀(t) is the baseline hazard function, X_MPR_ represents the calculated *Wasfaty* MPR value, X_Technique_ is a binary covariate (0 = critical errors present; 1 = perfect mastery), and X_Comorbidity_ is a binary covariate capturing the clinical control of mimic conditions (1 = managed or absent and 0 = unmanaged or active comorbidities). The regression coefficients (*β*₁, β₂, β₃) indicate the exact mathematical weight of each pharmacist-managed intervention on disease resolution. To maintain model symmetry and operational triage utility, the continuous X_MPR_ is dichotomized using a clinical threshold of 0.80 (X_MPR_ = 1 for MPR ≥80% and X_MPR_ = 0 for MPR < 80%). The baseline hazard function h₀(t) remains completely unspecified, establishing a semi-parametric framework that isolates behavioral impacts independent of the baseline time-to-control distribution shape. Before running the Cox proportional hazards model, the independence of all covariate streams, including the dichotomized binary MPR, qualitative device mastery, and clinical comorbidity parameters, was thoroughly verified using Variance Inflation Factors (VIF) derived from successive coefficient of determination *R*^2^*_k_* linear inversions (see Appendix A). All calculated values fell well below the conservative threshold of 2.5, proving that behavioral adherence, mechanical device technique, and unmanaged comorbidities function as independent clinical predictors within the SARP triage engine.

### 3.3 Systemic financial resource optimization equation

To provide the Ministry of Health and national insurance networks with a precise measure of the financial impact of the framework, the net systemic budget optimization is modeled via the following cost-avoidance formulation:

$$\Delta C_{\text{system}}=\sum_{j=1}^{M}[\begin{array}{l} (C_{\text{biologic},j}\times \omega_{j})-C_{\text{pharmacist}}\\ -C_{\mathrm{AOL}}-(C_{\text{exacerbation}}\times (1-\alpha_{j})\times \omega_{j}) \end{array}]$$

In alignment with the operational architecture of the SARP workflow (Figure 1), the administrative costs C_pharmacist_ and C_AOL_ are modeled as universal deductions applied across the entire cohort, reflecting their role as a mandatory gateway screening layer. Conversely, the residual risk penalty term comprising the acute emergency exacerbation cost C_exacerbation_ multiplied by the residual failure rate ( 1−α j) is conditionally restricted using the classification operator ω _j_. This ensures that emergency room downside risks are strictly billed to the behavioral optimization loop cohort, while true severe biological non-responders ω _j_ = 0 advance directly to targeted molecular initiation without artificial risk penalties.

Where Δ C_system_ represents the net financial savings achieved by the health network; *M* represents the total cohort size of uncontrolled difficult-to-treat patients systematically managed within the pathway; C_biologic, j_ is the projected annual cost of the targeted biological option for patient j; ω _j_ is a binary classification operator derived from the gatekeeper audit where ω _j_ = 1 if the patient is identified as non-compliant and diverted away from biologics, and ω _j_ = 0 if the patient passes all criteria and proceeds to treatment.

C_pharmacist_ is the operational cost of the pharmacy audit consultation session; C_AOL_ is the cumulative cost of administering the 12-week optimization infrastructure; C_exacerbation_ represents the average emergency acute stabilization cost incurred if a non-compliant patient suffers a major medical event; and α j represents the percentage reduction in acute exacerbations achieved through the pharmacist’s technique and adherence correction.

### 3.4 Addressing data limitations, ethical and cost precautions

Although both MPR and PCD provide scalable adherence tracking, they inherently measure medication acquisition rather than true consumption. To prevent patients from bypassing the gatekeeper through intentional medication dumping to artificially inflate their electronic scores, the methodological design mandates a physical reconciliation during the Tier 2 audit. The pharmacist is required to cross-reference the patient-reported usage patterns with the exact electronic timestamps generated by the *Wasfaty* platform. This dual-layer triangulation isolates intentional and unintentional non-adherence patterns from true physiological severe non-responsiveness with high precision, ensuring that advanced biological selections are predicated on verified behavioral patterns.

To ensure patient safety, the 12-week closed-loop is explicitly an active clinical accelerator rather than an administrative barrier. Patients routed into the Adherence Optimization Loop (AOL) are not subjected to therapeutic delays, rather, they receive immediate interventions designed to correct modifiable execution errors. Furthermore, this operational design accounts for the added administrative and workforce costs associated with running these clinics by explicitly embedding the costs of pharmacy consultations (C _pharmacist_) and optimization loop management (C _AOL_) directly into the systemic financial optimization model. Because targeted biological therapies present a massive recurring financial liability, the non-recurring operational cost of pharmacist triage sessions yields an exceptionally high, stable Return on Investment (ROI). This layout balances immediate health network expenditures with long-term macroeconomic resource sustainability.

## 4 Policy implications and workforce evolution

### 4.1 Eradicating reporting bias through digital phenotyping

The integration of centralized e-prescribing data into the criteria for severe asthma effectively dismantles the deep-seated methodological limitations that have historically plagued respiratory routine practice, namely, Social Desirability Bias and the Hawthorne Effect. Social Desirability Bias represents a behavioral phenomenon where patients systematically over-report adherence and mask execution failures during interviews due to an intrinsic desire to satisfy healthcare providers or avoid the social stigma associated with non-adherence. Similarly, the Hawthorne Effect is a temporary behavioral shift towards improving adherence solely because of the awareness of being actively monitored (27, 28). In conventional clinical pathways, clinicians are trapped within a dense fog caused by an over-reliance on subjective monitoring tools such as patient self-reporting, interviews, and retrospective diaries. These modalities are heavily compromised by active recall failure and social desirability biases, causing systemic overestimation of actual medication usage, which can lead to premature treatment escalation (18). Furthermore, patients frequently exhibit temporary adherence acceleration immediately preceding a scheduled clinical appointment, a phenomenon that masks chronic, sub-optimal controller utilization.

### 4.2 Triage of non-adherence phenotypes

SARP introduces objective passive digital phenotyping and provides an unvarnished, longitudinal audit of true medication acquisition behavior. While the MPR serves as a robust proxy for behavior, it measures medication acquisition rather than direct consumption.

SARP ingeniously resolves this limitation by pairing the quantitative *Wasfaty* digital audit with a mandatory, face-to-face physical technique assessment conducted by a pharmacist. This dual-layer triangulation effectively isolates intentional and unintentional non-adherence patterns from genuine physiological non-responsiveness. By categorizing these behaviors into distinct clinical phenotypes, the Adherence Optimization Loop (AOL) deployed within SARP applies targeted behavioral and operational countermeasures rather than non-specific clinical escalations (Table 2).

**Table 2:** Behavioral phenotypes of non-adherence and corresponding intervention strategies.

Adherence phenotype	Underlying drivers	Typical clinical presentation	Targeted intervention strategy
Unintentional non-adherence	Forgetfulness, competing priorities, regimen complexity, poor inhaler technique, limited treatment knowledge	Patients willingness to adhere but demonstrate inconsistent medication use or incorrect inhaler handling	Digital reminders, regimen simplification, pharmacist-led inhaler technique training, structured educational interventions
Intentional non-adherence	Negative treatment beliefs, fear of adverse effects, perceived lack of benefit, medication skepticism	Deliberate dose reduction, intermittent use, or treatment discontinuation despite prescription availability	Shared decision-making, motivational interviewing, individualized risk–benefit counseling, health literacy interventions
Complex or multifactorial non-adherence	Combined perceptual, behavioral, social, psychological, and structural barriers	Persistent poor adherence; mental health disorders, social deprivation, or multimorbidity	Multidisciplinary integrating pharmacy, behavioral health, social support, and intensive management

### 4.3 Advanced pharmacy practice and return on investment (ROI) under Saudi vision 2030

The operational execution of the SARP framework highlights a profound comparative advantage inherent to the modernized Saudi Arabian healthcare ecosystem. While prominent international health networks such as the National Health Service (NHS) in the United Kingdom or the Veterans Affairs (VA) network in the United States have long recognized the value of using pharmacy data to optimize clinical outcomes, their efforts are chronically bottlenecked by systemic data fragmentation. In these international systems, patients routinely transition between disparate private, corporate, and municipal pharmacy providers. This fragments their longitudinal dispensing records, compromises multi-center data collection, and creates extensive blind spots in adherence monitoring frameworks. In contrast, the sweeping digital transformation driven by Saudi Vision 2030 has unified all public and private pharmacy dispensing channels under a single, centralized cloud database architecture via the *Wasfaty* platform. This structural centralization transforms the Kingdom into a premier global living laboratory for value-based digital medicine. SARP capitalizes on this infrastructure to build a closed-loop system, establishing an international blueprint for population-scale biologic stewardship in precision respiratory care.

### 4.4 Economic stewardship and workforce transformation

As the Saudi Health Sector Transformation Program shifts toward the deployment of Accountable Care Organizations (ACOs), demonstrating a clear Return on Investment (ROI) for clinical workflows has become an institutional necessity. The economic implications of the digital gatekeeper model are profound. Misclassifying even a small fraction of non-adherent, difficult-to-treat patients as severe asthmatics can result in massive systemic resource inflation to pharmacologically override a behavioral problem. SARP simultaneously will drive a critical workforce transformation, expanding advanced pharmacy practice integration across care networks. National literature demonstrates that a substantial clinical gap exists. Approximately 56.4% of patients with asthma receive insufficient home management education, and 26.6% demonstrate critical inhaler technique errors. By redefining the pharmacist’s role from a supportive educator into an authorized clinical auditor, the health system implements a highly reliable triage mechanism. This preserves specialized tertiary care slots exclusively for patients of high physiological complexity.

### 4.5 Limitations and future directions

We acknowledge that while the combination of *Wasfaty* tracking and live pharmacist technique audits minimizes medication stockpiling and dumping, further precision can be achieved. Future iterations of the SARP framework should incorporate Electronic Monitoring Devices (EMDs) such as smart inhaler sensors that track real-time lung deposition and inhalation synchronization. Integrating EMDs via the national *Sehaty* API would establish a seamless ladder. Additionally, pairing these data streams with objective point-of-care clinical biomarkers, such as fractional exhaled nitric oxide (FeNO) and blood eosinophil profiles, will allow for real-time tracking of treatment efficacy. This approach will lay the foundation for a highly sophisticated, data-driven national severe asthma registry.

To bridge current operational gaps and achieve the value-based objectives of Saudi Vision 2030, we propose the following strategic framework for nationwide deployment:

- Mandating *Wasfaty* adherence reports for biologic pre-authorization
  The Ministry of Health (MOH) should integrate a mandatory data validation field within all digital prior-authorization portals. Approval for any specialized respiratory biologic must be programmatically locked until a verified, 90-day *Wasfaty* report proving an MPR ≥ 80% is securely appended to the electronic case file.
- Establishing standardized institutional codes for pharmacist adherence clinics
  To ensure clinical scalability and formal visibility within individual hospital networks, specific billing codes and dedicated electronic health record (EHR) slots must be institutionalized for “Pharmacist-Led Adherence Clinics.” These clinics will function as a compulsory secondary filter, managing and thinning the referral pipeline directed toward tertiary care consultants.
- Pioneering the Saudi national severe asthma digital registry
  We call for the creation of a real-time, nation-wide Severe Asthma Registry hosted on the national health cloud infrastructure. By programmatically linking baseline molecular biomarkers (FeNO, blood eosinophil counts, serum IgE) with continuous *Wasfaty* behavioral telemetry, the Kingdom can establish definitive, large-scale datasets evaluating long-term biologic efficacy, clinical remission rates, and true macroeconomic cost-effectiveness.

## 5 Conclusion

Differentiating between modifiable pseudo-severe (difficult-to-treat) and true severe asthma represents a critical operational, clinical, and financial milestone for contemporary health systems. Continuing to rely on unverified, subjective patient tracking keeps clinics operating in a fog that compromises patient safety and exhausts valuable public health funding. The proposed digital gatekeeper model addresses this challenge by turning Saudi Arabia’s unique centralized digital landscape into an active tool for precision respiratory care. Mandating data-driven adherence protocol tracking cycle through the *Wasfaty* platform ensures that advanced biological therapies are reserved for patients with confirmed physiological phenotypes. This framework delivers a reproducible, world-class model of resource stewardship and clinical excellence, advancing the core healthcare transformation mandates of Saudi Vision 2030.

## Data Availability

No raw data was generated in the preparation of this manuscript.
