# Supplementary material for: The digital gatekeeper: a practical operational framework for differentiating pseudo-severe from severe asthma
Source: Front Med (Lausanne). 2026 Sep 8;13:1909321. doi: 10.3389/fmed.2026.1909321 (PMC13595366; doi:10.3389/fmed.2026.1909321)
Supplement: Supplementary file 1 [file Data_Sheet_1.pdf]

Appendix A

Variance Inflation Factor (VIF) Calculation Framework for Covariate Independence Verification

Objective and Background Matrix

Before execution of the semi-parametric Cox Proportional Hazards regression model h(t|X), standard clinical model validation requires the verification of the assumption of non-multicollinearity among the independent predictor variables. This appendix outlines the strict mathematical sequence used to derive the individual Variance Inflation Factors (VIF_k) for the three primary covariates within the Saudi Asthma Referral Pathway (SARP) triage engine:

1. ***X_1 (X_{MPR})***: Binary Medication Possession Ratio (1 = Good Adherence ≥80%, 0 = Poor Adherence < 80%).
2. ***X_2 ($X_{Technique}$)***: Binary Inhaler Technique Mastery (1 = Perfect Mastery, 0 = Critical Errors).
3. **X_3 ($X_{Comorbidity}$)**: Binary Mimic Conditions Status (1 = Managed/None, 0 = Unmanaged/Active).

Step 1: Formulation of Auxiliary Linear OLS Regressions

To calculate the unique collinearity impact of each covariate, the statistical tracking framework isolates each predictor sequentially, treating it as a dependent variable while setting the remaining variables as simultaneous linear regressor parameters. This establishes three separate auxiliary ordinary least squares (OLS) equations:

$$\text{Equation 1: } X_1 = \gamma_{1,0} + \gamma_{1,2}X_2 + \gamma_{1,3}X_3 + \epsilon_1$$

$$\text{Equation 2: } X_2 = \gamma_{2,0} + \gamma_{2,1}X_1 + \gamma_{2,3}X_3 + \epsilon_2$$

$$\text{Equation 3: } X_3 = \gamma_{3,0} + \gamma_{3,1}X_1 + \gamma_{3,2}X_2 + \epsilon_3$$

Where $\gamma_{k,i}$ represents the linear regression coefficients for the auxiliary matrices, and ε_k accounts for the standard independent error terms.

Step 2: Extraction of Coefficients of Determination R^2_k

For each auxiliary regression executed across the *M*-patient study cohort, the software extracts the unadjusted coefficient of determination (*R^2_k*). The parameter R^2_k reflects the exact proportion of variance in covariate X_k that can be linearly predicted and explained by the combination of the alternative indicators:

$$R_k^2 = 1 - \frac{\text{RSS}_k}{\text{TSS}_k} = 1 - \frac{\sum_{j=1}^{M}(X_{j,k} - \hat{X}_{j,k})^2}{\sum_{j=1}^{M}(X_{j,k} - \bar{X}_k)^2}$$

Where RSS_k represents the residual sum of squares, TSS_k defines the total sum of squares, $\hat{\mathbf{X}}_{j,k}$ is the predicted model estimate for patient j, and $\bar{X}_k$ represents the categorical cohort mean value.

Step 3: Execution of the VIF Inversion Formula

The variance of an estimated regression coefficient is inflated by a factor derived directly from the linear overlap value. The final calculation computes the scalar inflation value by applying the standard inversion formula to the remaining tolerance profile $(1 - R^2_k)$:

$$\text{VIF}_k = \frac{1}{1 - R_k^2}$$

- **Mathematical Boundary Check**: As $R^2_k \to 0$ (perfect orthogonal independence), $\text{VIF}_k \to 1$, indicating absolutely zero standard error inflation. Conversely, if $R^2_k \to 1$ (perfect collinear overlap), $\text{VIF}_k \to \infty$, rendering estimated beta parameters completely unstable.
- While the independent parameters are structurally dichotomized, standard linear VIF diagnostics remain highly robust and mathematically conservative for identifying destabilizing multicollinearity loops. Given that all observed calculations fall substantially below the strict 2.5 boundary threshold, the potential for non-linear collinear distortion is effectively minimized, ensuring stable standard errors and unbiased log-hazard estimations within the primary pathway.

Validation and Threshold Mapping

In standard biomedical and health-economic literature, a strict threshold profile is applied to verify baseline parameter stability: Maximum Tolerance Boundary Cutoff: $\text{VIF}_k < 2.5$

To evaluate the mathematical boundary constraints represented by the study's verified finding ($\text{VIF}_k < 2.5$), the cross-sectional linear parameters can be backwards solved exactly to determine the maximum permissible data leakage:

$$2.5 = \frac{1}{1 - R_k^2} \Longrightarrow 1 - R_k^2 = 0.40 \Longrightarrow R_k^2 = 0.60$$

This step proves that less than 60% of the underlying variance in any single behavioral indicator is shared with or explained by the other clinical elements. Because the real software calculations fall well below this 2.5 boundary, standard errors remain completely un-inflated, and individual β coefficients within the main Cox pathway can be trusted as unbiased indicators of clinical speed-to-remission.
